# Study on the Measurement of Laser Drilling Depth by Combining Digital Image Relationship Measurement in Aluminum

**DOI:** 10.3390/ma14030489

**Published:** 2021-01-20

**Authors:** Chao-Ching Ho, Guan-Hong Li

**Affiliations:** 1 Graduate Institute of Manufacturing Technology, National Taipei University of Technology, Taipei 10608, Taiwan; 2 Mechanical Engineering, National Yunlin University of Science & Technology, Douliu 64002, Taiwan; M10211050@yuntech.edu.tw

**Keywords:** digital image correlation, laser processing, depth measurement

## Abstract

In this study, laser processing equipment was used to drill aluminum alloy materials and with different auxiliary mechanisms, the deformation around the holes after processing was observed. The experimental results show that, due to the high temperature generated during laser processing, a large thermal gradient causes thermal stress to be introduced into the test piece and outward expansion deformation occurs. In this study, the digital image correlation and residual stress detection methods were applied. Based on the correlation between the drilled hole depth and the hole deformation, the hole depth of the laser processing was estimated. The average coefficient of determination for all auxiliary mechanisms is 0.82. The experimental results confirm that the digital image correlation method can be used to estimate the hole depth of laser processing.

## 1. Introduction

Aluminum 5052 is widely used in industrial applications owing to its material properties of lower melting points [1], lightness [2], high fatigue strength [3], and corrosion resistance in maritime environments [4]. Among the machining operations, the drilling process of aluminum alloys is the most challenging [5] and the fatigue life of industrial components depends on the quality of drilled holes [6]. Aluminum alloys are difficult-to-laser-drilling materials due to their high reflectivity [7]. Furthermore, high thermal conductivity of aluminum alloys tends to broaden more regions of the heat-affected zone with laser beam machining [8]. McNally et al. reported that the quality of the laser drilled holes and the accuracy of the laser drilling process are of utmost significance for the manufacture of aeroengine parts [9]. Wang et al. reported a quality control diagram for precise laser drilling on metal materials [10].

Laser drilling depth measurement is a significant issue in industrial applications [11]. The hole depth is the important process and performance parameter in laser beam drilling process [12]. The earliest methods used contact for measurements and, subsequently, a number of global, non-contact measurement methods were adapted to different detection environments. Among the non-contact deformation measurement methods, optical measurement is most widely used [13]. Our previous work used coaxial machine vision to perform breakthrough detection [14] and control deep hole processing [15]. Ding et al. developed a laser power monitoring and control system based on the digital optical comple mentary metal-oxide semiconductor (CMOS) sensor [16]. In 1982, Peters and Ranson et al. [17] proposed using the characteristics of digital images to observe the deformation of objects after they were subjected to force, generating interference fringes on the surface of the object to be tested by laser light, and using a camera to capture the deformation of the object before and after deformation. The concept of calculating the amount of displacement via images is expected to lead to the further development of digital image correlation (DIC) measurement technology in the future. In 2000, Dıaz et al. [18] used digital speckle pattern interferometry to detect the strain release near the hole after drilling a test piece with residual stress. However, the optical system cannot be easily set up in the interferometry method, which is also affected by environmental shocks. In 2009, Hatalkeh et al. [19] used X-ray diffraction to measure the stress value of the welded part subjected to laser heating via a tensile test. Although the test environment can be easily established, X-ray diffractometers are expensive. Thus, this approach remains unaffordable for many applications and is limited to measurements applied to shallow layers. In 2004, Dr. Shao Yaoxian of the National Tsinghua University [20] studied the thermal strain behavior of workpieces during laser drilling using numerical analysis, and explored the behavior using experimental methods. In 2006, D. Lecompte and other scholars [21] used the same test strip to apply three different camera magnifications, using image morphology to identify the size distribution of random speckles and different subset sizes. The DIC calculations showed that larger size random speckles combined with large reference subsets could yield more accurate displacement data, indicating that DIC does not have to use the most detailed spots to obtain accurate results [22]. To obtain the most accurate data, appropriate matching of the size of the reference subset is required.

In 2012, Song et al. [23] used the DIC method and focused ion beam milling to investigate the residual stress values of films with submicron thickness. First, the focused ion beam processing method was used to produce the surface of the object to be tested. Several circular hole arrays were used to determine the random spots of displacement by the DIC method, and a square groove was then machined around the holes. The experimental results showed that the strain release inside the square groove increases with the depth of the groove depth. Furthermore, the depth is proportional to the square groove length. The fully released strain value, as the lower value of the residual stresses, is released as incremental cutting progresses. In 2018, T. Mikołajczyk et al. [24] demonstrated the potential of combining image recognition software and Artificial Neural Network (ANN) modeling for tool life estimation in a machining process. The rapid advancement in digital image processing highlights the potential for machine vision to be used in the field of machining.

This study demonstrates the feasibility of the DIC method to measure residual stress on a small scale. In this work, the laser processing method was used to drill an unstressed test piece. Section 2 describes the use of DIC to detect surface deformation in engineering materials. Various auxiliary laser-processing mechanisms were used to observe the deformation of aluminum around the hole after machining. The experimental mechanisms were divided into unassisted laser processing and a vortex jet-assisted laser. Different auxiliary mechanisms, i.e., vortex jet-assisted laser processing, a test strip preheating mechanism, and surface application of thermal paste, were employed to change the temperature gradient on the surface of the test piece during laser processing, thereby affecting the generation of thermal strain. When performing DIC, the reference subset affects the accuracy of the calculation result, and the image processing method was thus used to find the largest spot size inside the calculation area. When the reference subset was larger than the maximum spot size, the obtained displacement distribution field was closer to the actual deformation of the test piece. Section 3 presents the investigation of the subset size, which was chosen to enable the measurement sensitivity to monitor the surface deformation caused by laser machining. Furthermore, the difference between the maximum and minimum displacement values around the hole was measured by DIC to judge the advantages and disadvantages of different machining aids and to estimate the hole depth of laser processing.

## 2. Materials and Methods

The laser drilling of a surface hole on a specimen induces a local thermal strain release that generates a displacement field in the direction perpendicular to the drilled plane. To investigate the thermal effect induced by laser drilling, the measurement of thermal strain is significant. The digital image relationship method is a full-area, non-contact type of strain measurement method. After the test piece is processed and deformed, the imaging device is used to capture the image before and after the surface deformation of the test piece. Each pixel in the image has a gray level. The DIC method compares the gray levels of two images before and after deformation, and determines the correlation between the two images after numerical calculation so that the displacement distribution field of the whole region, and the displacement distribution field, are obtained. After numerical calculation, the displacement distribution field is converted into a strain distribution field.

### 2.1. Reference Subset

**Figure 1:**
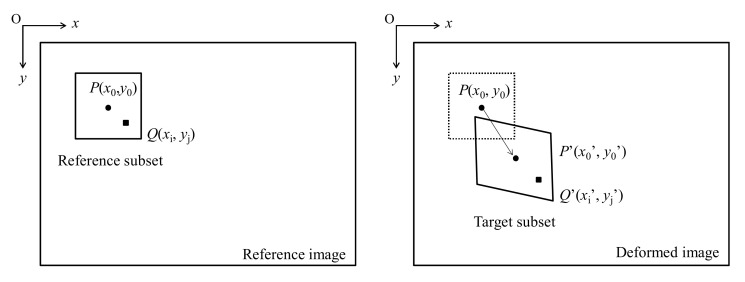
Reference subset before deformation and target after deformation.

As shown in Figure 1, the center point *P′*(*x_0_′*, *y_0_′*) represents the deformed position. For the position mapped by the arbitrary point *Q*(*x_i_*, *y_j_*) in the reference subset, the change in the X- and Y-directions of the coordinate can be obtained using the DIC method.

### 2.2. Related Functions

To evaluate the degree of similarity between the pre-deformation and post-deformation subsets, the gray value distribution of the reference and target subsets must be analyzed using the cross-correlation function, and calculated by the interaction correlation function. The correlation coefficient is used to judge the degree of agreement. The definitions of the related functions are slightly different and can be divided into two categories: the cross-correlation criterion and the sum of squared differences criterion.

### 2.3. Experimental Setup and Method

**Figure 2:**
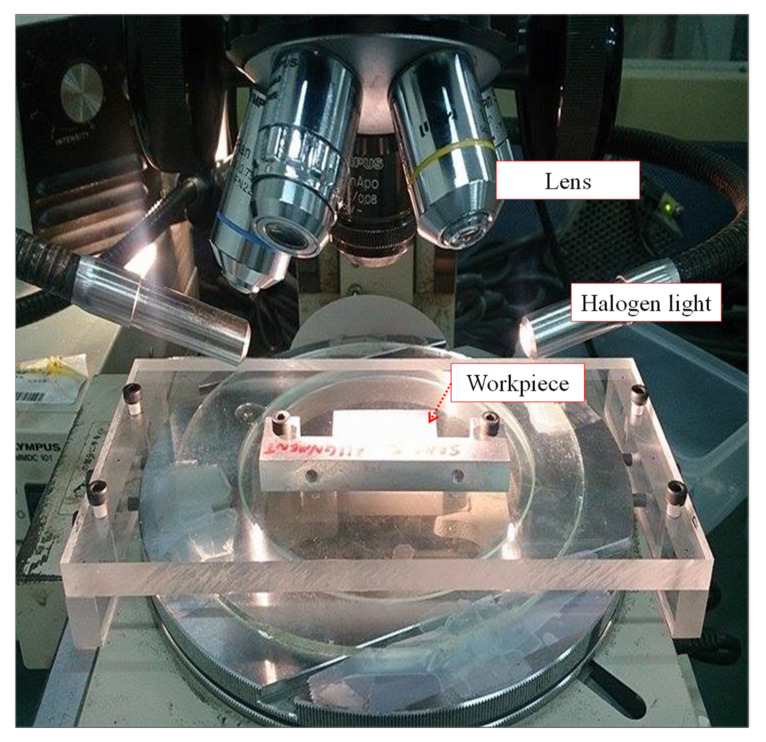
OLYMPUS U-PMTVC 8C14561 tool microscope.

Calibration was performed before the DIC measurement to minimize the variance. In this paper, an OLYMPUS microscope (Figure 2) (Olympus, Tokyo, Japan) was used to capture the image before and after processing. A charge coupled device (CCD) camera (U-PMTVC 8C14561, Tokyo, Japan) above the microscope with a 2.5× objective lens was used to capture images with a magnification of 25×; the field of view was about 2.6 × 1.95 mm^2^, and the image size was 640 × 480 pixels. A simple positioning platform was added to the XY platform to align the test piece with the positioner, thus ensuring that the position of the processed image was the same after the test piece was processed. A halogen light source was used as a light-emitting device, and the XY stage was used to move the field of view to the position to be processed for shooting.

**Figure 3:**
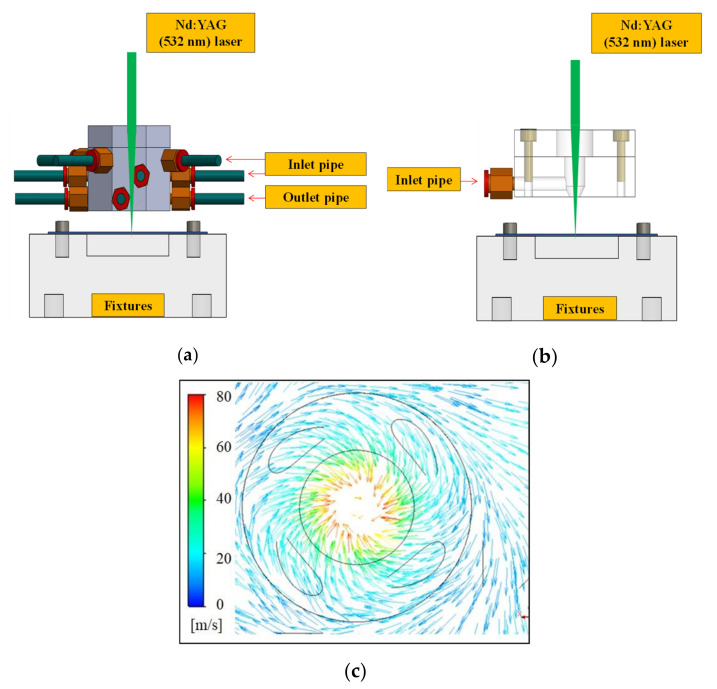
Schematic diagram of two gas-assisted mechanisms: (**a**) vortex jet assist mechanism; (**b**) direct injection auxiliary mechanism; (**c**) flow line distribution on the surface of the test piece.

In addition to pure laser processing, the experiment used four auxiliary mechanisms developed in our previous works [25,26]. The first auxiliary mechanism uses the direct injection mechanism, the second auxiliary mechanism uses a vortex jet mechanism to assist the laser drilling, and the third auxiliary mechanism is the use of heating. The test piece was preheated. The laser machining parameters of the experiment are listed in Table 1. The air jet pressure is listed in Table 2. Experiments were conducted using 6 mm-thick plates of aluminum (Al 5052) sheet. The chemical compositions of Al 5052 were Al 96.6%, Mg 2.2%, Fe 0.4%, Cr 0.2%, Si 0.2%, Mn 0.1%, Zn 0.1%, Cu 0.1%, and other elements 0.1%. The mechanical properties of Al 5052 were an ultimate tensile strength of 218 MPa and micro hardness of 89 HV. The last auxiliary mechanism was the application of thermal grease to the surface of the test piece. Figure 3 shows a schematic diagram of two gas-assisted mechanisms. Figure 3a shows a vortex jet assist mechanism, for which the overall material is stainless steel. The structure consists of four air intake pipes and eight exhaust pipes. An ML-808 FX dispenser is connected to the intake pipe to provide jet airflow, and a Rocker 300 oil-free vacuum pump is connected to the exhaust pipe to provide a pumping function. When the machine is started, a swirling airflow is generated under the mechanism and on the surface of the test piece. Figure 3c shows the flow line distribution on the surface of the test piece. It can be seen that when the swirling mechanism is activated, an upward airflow forms on the surface of the test piece. In the laser processing process, this can help with removal of slag chips and accelerate the efficiency of laser drilling. Figure 3b shows the direct injection auxiliary mechanism, which is relatively simple and made of acrylic. Only one air inlet pipe is connected to the ML-808 FX dispenser (Musashi, Tokyo, Japan). When the dispenser is started, air is sprayed onto the surface of the test piece. The slag can be directly sprayed out during the processing.

## 3. Results and Discussion

### 3.1. Selected Reference Subset of Digital Image Correlation (DIC)

When using digital image correlation (DIC) to calculate the displacement distribution, several factors affect the accuracy of the calculation, particularly the size of the area spot and the size of the reference subset, because digital image correlation (DIC) measures the brightness of all of the pixels in the subset. The value is used as the feature matrix to perform the correlation matching operation. Therefore, the reference subset must have sufficient features, otherwise the calculated displacement distribution data generate a large error. When the displacement distribution is close to that of the real situation, a reference subset that is too small may result in the situation in which the displacement cannot be resolved; conversely, if the reference subset size is too large, operation time increases. Thus, determination of the size of the reference subset is an important issue. The reference subset size has a particular relationship with the size of the speckle spot, which is why this study used image geometry to measure the spot size inside the DIC calculation area in the image.

#### 3.1.1. Obtaining Spot Size by Image Processing

**Figure 4:**
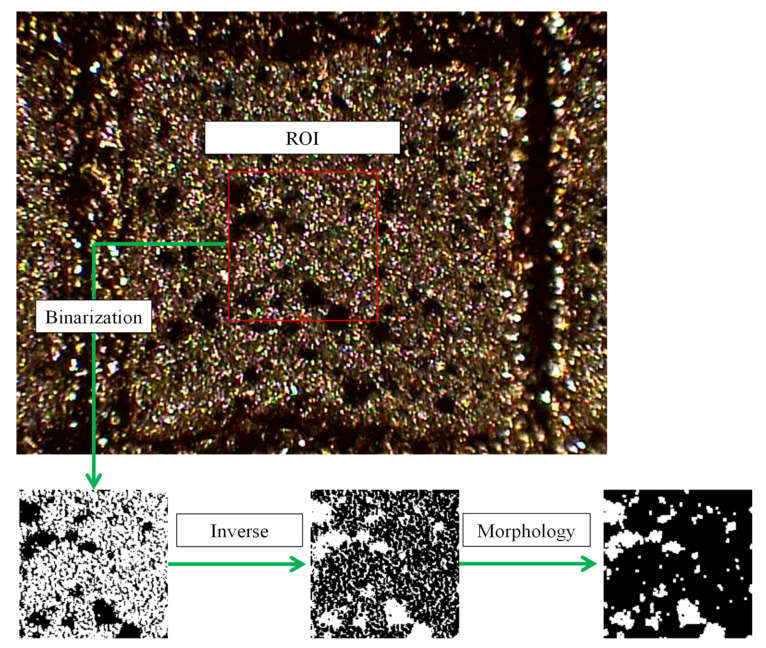
Results of stepwise processing using image graphics.

The calculated samples were randomly selected from three sets of images before and after the deformation in the electrical discharge machining experiment. The initial step of image processing performed image binarization of the region of interest (ROI). The binarization process was performed by comparing gray values with a threshold and the image data converted into just values of black and white. After the binarization process, the gray levels were inverted to act as a mask region to remove noise. Invert operation interchanged the black region to white and vice versa. Then, the morphological processing of the binary result improved speckle identification. Morphological operation was a technique of image processing that shrunk and expanded specific shapes contained in the input binary image. The morphological operation was performed with the erode operation followed by the dilate operation. Figure 4 shows the results obtained by each procedure in the image processing process. Table 3 shows the maximum spot size obtained after image morphology of the three groups of samples.

#### 3.1.2. Correlation between Reference Subset and Spot Size

**Figure 5:**
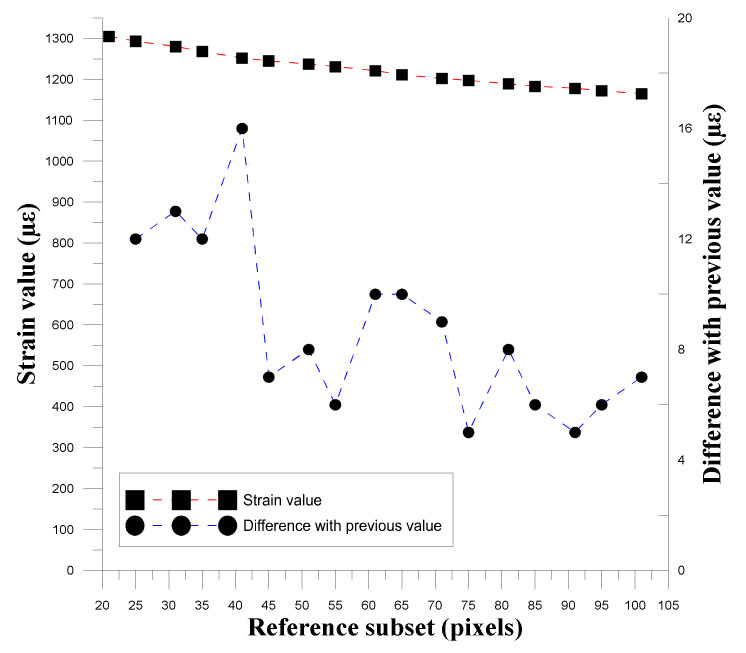
Trend graph of strain change and strain difference of the first set as the size of the reference subset changes.

**Figure 6:**
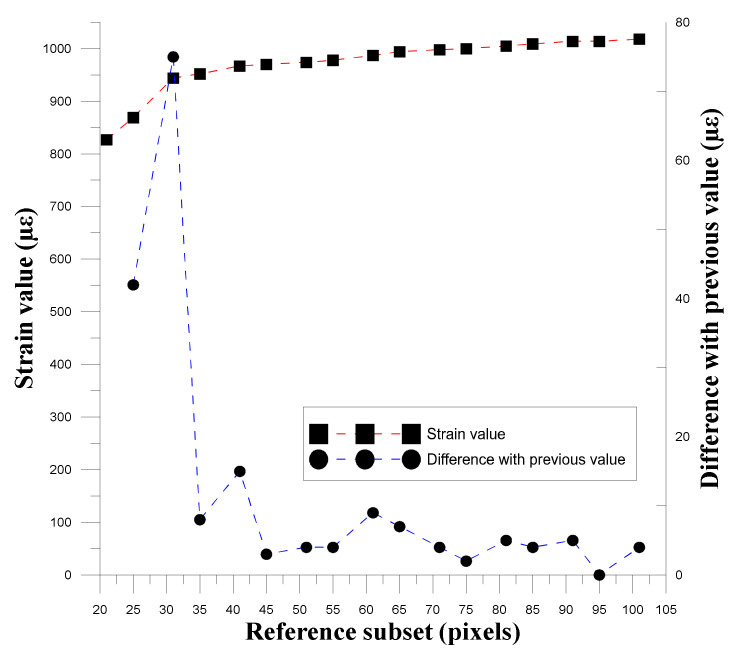
Trend graph of strain variation and strain difference in the second group as the size of the reference subset changes.

**Figure 7:**
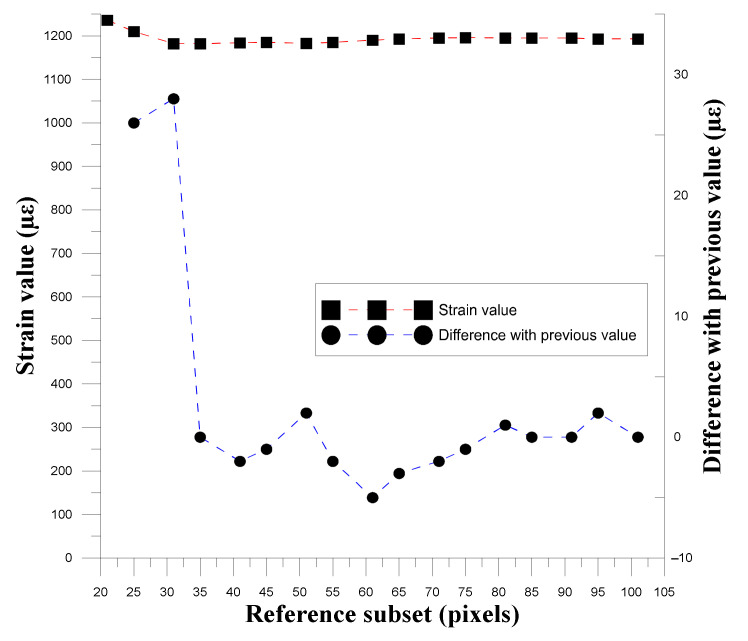
Trend graph of strain variation and strain difference in the third group as the size of the reference subset changes.

To confirm the relationship between the reference subset size and the spot size, this study used different reference subsets to calculate and convert the strain values, and used the obtained strain values to observe the relationship between the spot size and the degree of strain. ∆ε was used to determine the changes produced by the gradual expansion of the reference subset, as shown in Equation (1), and Table 4 shows the reference subset used.

(1) $$∆\varepsilon =\left|\varepsilon_{M_{i}}-\varepsilon_{M_{i+1}}\right|,\;i\;=\;1,\;2,\;3,\;\ldots ,\;17$$

where $\varepsilon_{M_{i}}$ and $\varepsilon_{M_{i+1}}$ are the strains of the displacement data conversion for the DIC calculation using the reference subset. Figure 5, Figure 6 and Figure 7 show the relationships between the three experimental reference subsets and the strain. It can be seen that the first set of data was calculated from the strain value calculated by the reference subset size of 41 pixels minus the strain calculated by 35 pixels. The ∆*ε* value obtained was the largest. In the second and third sets of data, the ∆*ε* value obtained by subtracting the strain value calculated by 25 pixels from the reference subset size of 31 pixels was the largest. The subsequent ∆*ε* values were much smaller than the maximum value, thus verifying that when the reference subset size is close to the spot size, displacement distribution data close to the actual situation can be obtained.

### 3.2. Laser Drilling with Displacement Measurement of Different Auxiliary Mechanisms

**Figure 8:**
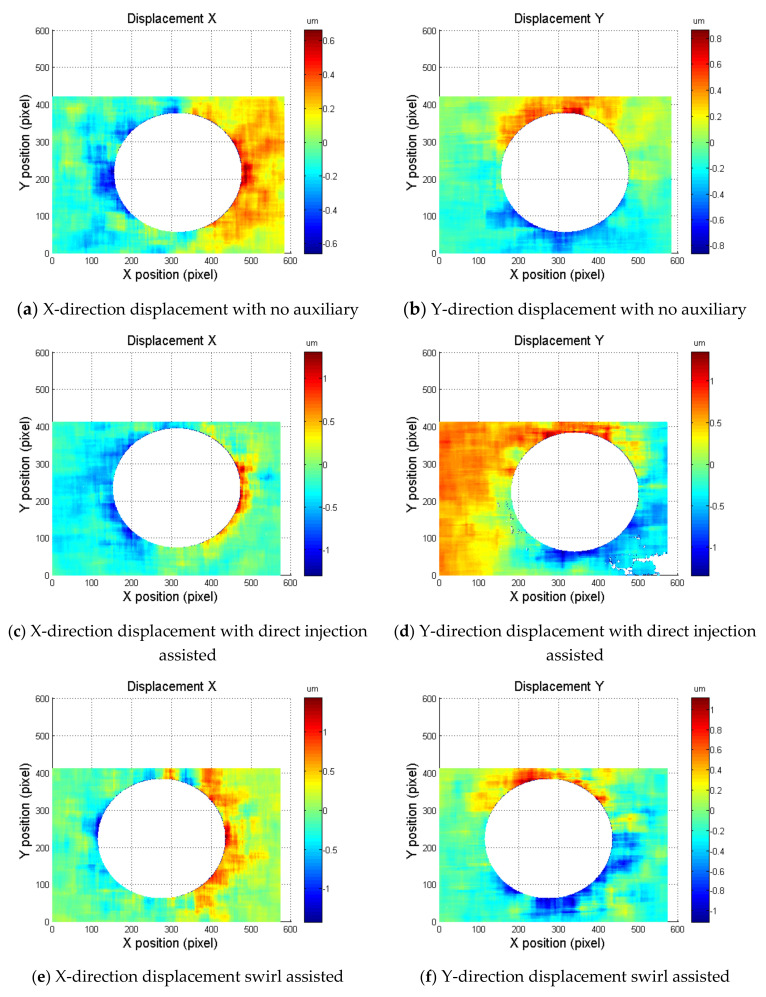

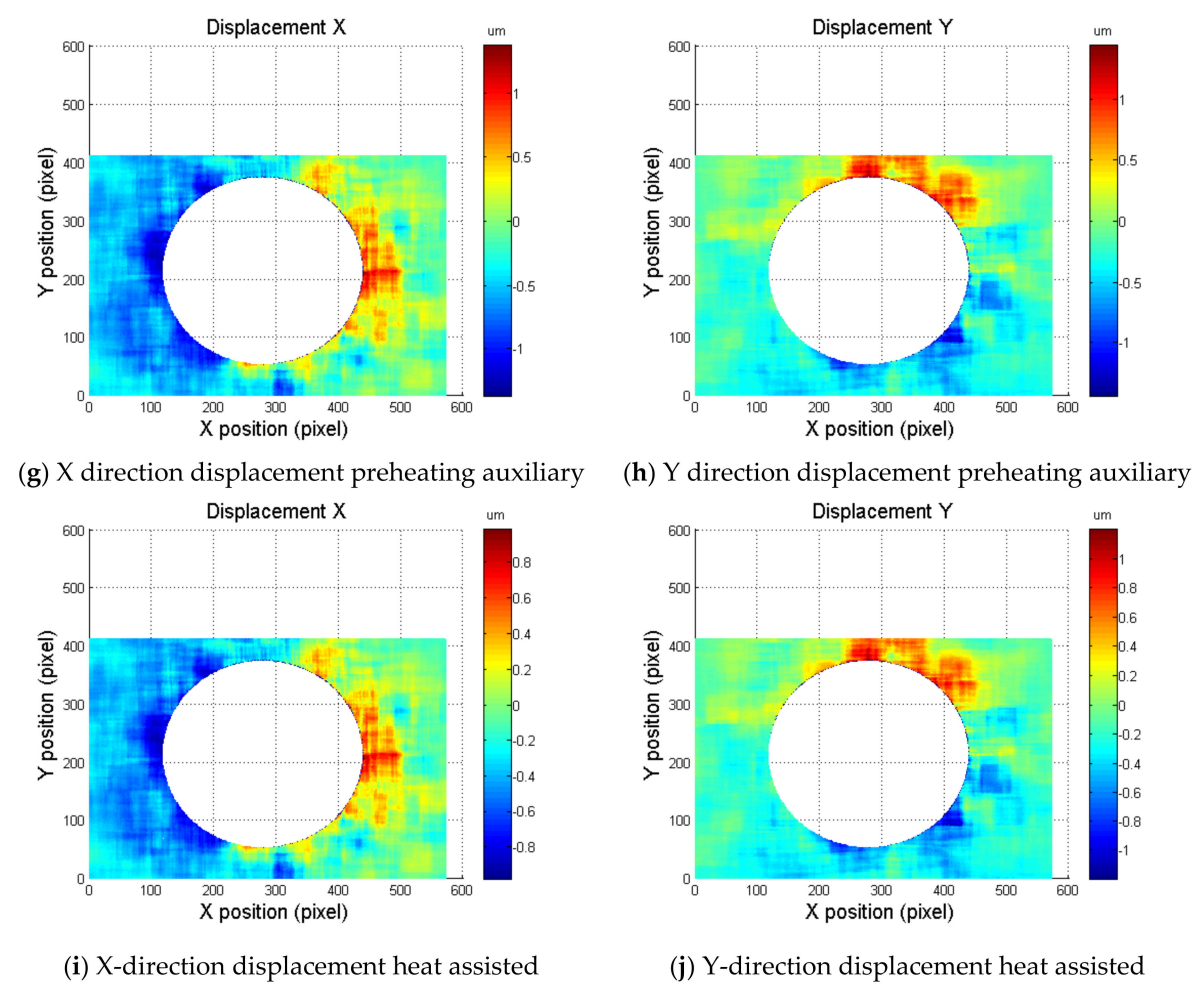
The measured surface deformation under different assist mechanisms.

The DIC method was applied to characterize the thermal effect produced by laser drilling for aluminum alloy Al5052 under different assisted laser drilling approaches. The laser drilling parameters are listed in Table 1. The implementation of the displacement measurement of different auxiliary mechanisms consists of the following consecutive steps: (1) To obtain precise determination of the positions of the specimen for the reference image, a pilot hole was drilled. (2) During the DIC analysis of the captured images, a square area with a size of 161 × 161 pixels in the middle of the specimen was chosen as the region of interest. After capturing the DIC reference image, different assisted laser drilling methods were used to produce residual thermal strain by drilling a hole in the region of interest. The DIC image of the area around the drilled zone was then captured and analyzed. The acquired images were processed using a self-developed DIC program to obtain the surface deformation. Figure 8 shows the displacement distribution of the X-direction and Y-direction of the test piece after laser drilling with different auxiliary assisted methods. The center of the machining displacement was deducted from the diameter of 160 pixels (about 650 μm), and the X-direction can be seen. In the displacement map, the left side of the hole is red (moving in the +X direction) and the right side of the hole is blue (moving in the −X direction). In the displacement distribution in the Y direction, the hole is red (moving in the +Y direction) and blue under the hole (moving in the −Y direction). This proves that the material near the hole after laser drilling has a very high temperature gradient, which causes the test piece to experience thermal stress.

**Figure 9:**
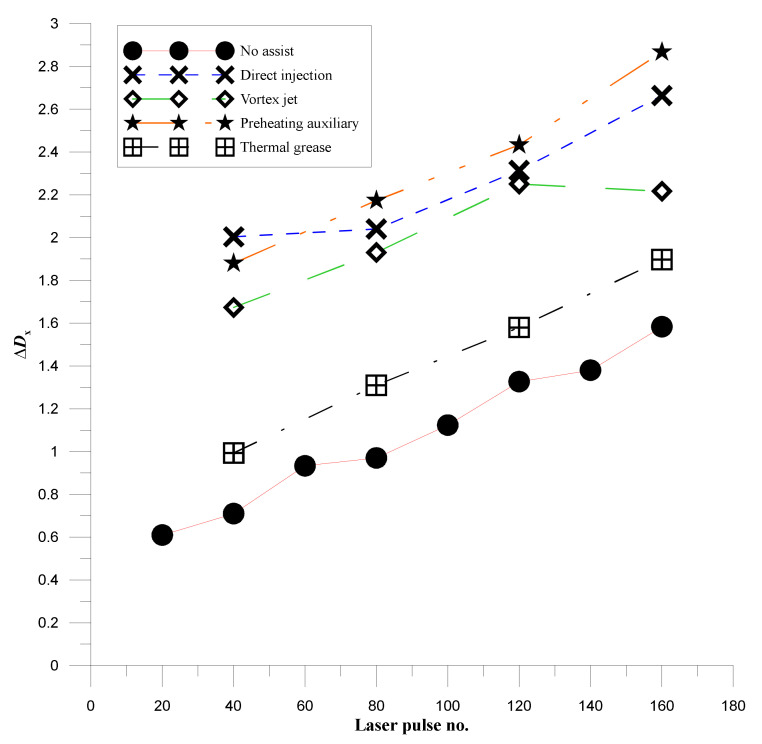
Curves of laser emission and deformation difference for laser processing with different auxiliary mechanisms.

To discuss the amount of deformation around the hole under different numbers of hairs, Equation (2) was used to obtain the difference between the maximum and minimum deformations around the hole ∆*D_x_*:

(2) $$∆D_{x}=dispX_{max}-dispX_{min}$$

where $dispX_{max}$ is the maximum value of the X-direction displacement distribution data and $dispX_{min}$ is the minimum value of the X-direction displacement distribution data. Figure 9 shows the laser emission number and deformation gap curve of laser processing with different auxiliary mechanisms. From the curve distribution map, it can be seen that ∆*D_x_* increases with the increase in the number of hairs when no auxiliary mechanism is used for laser processing (red curve), which proves that the lightning generates a significant thermal gradient to introduce thermal stress. The greater the accumulation, the greater the deformation of the auxiliary part of the thermal paste, because the heat dissipation rate is faster than in the case with no assistance. This slightly increases the temperature gradient of the test piece, resulting in greater deformation. Because the gas is directly sprayed on the surface of the workpiece, the temperature change is accelerated by gas cooling. The thermal gradient of the surface of the workpiece is increased, and the pressure of the jet gas also induces shear stress in the workpiece, thereby causing greater deformation around the hole. The preheating auxiliary part can reduce the temperature gradient during processing after raising the temperature of the workpiece, but a swelling effect occurs after the workpiece is heated, so the calculation of deformation of the test piece also includes noise, and the deformation results in a hole that is too large.

**Figure 10:**
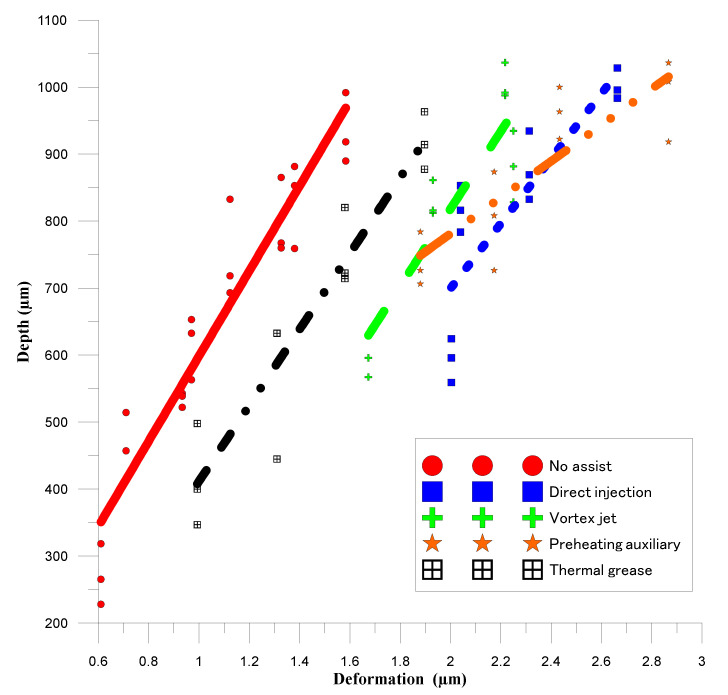
Relationship between hole deformation and hole depth in laser processing with five auxiliary mechanisms.

**Figure 11:**
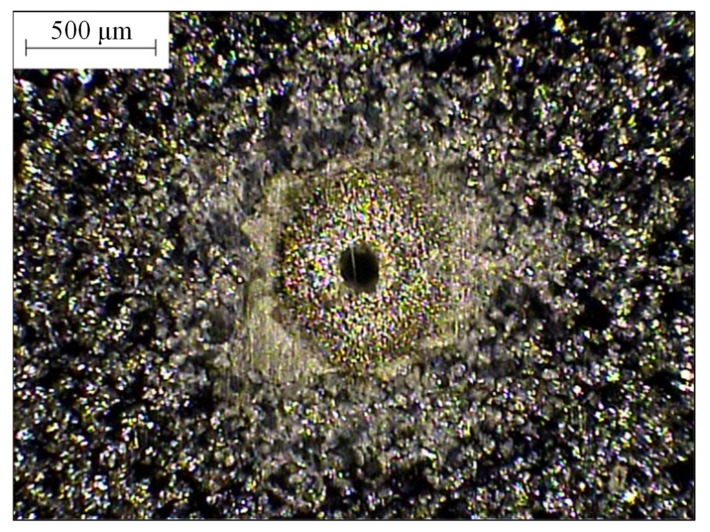
The actual image of the drilled hole.

Figure 10 shows the relationship between the hole deformation and the hole depth of laser processing using five auxiliary mechanisms. Linear regression indicated the coefficient of determination of 0.91 (no assistance), 0.90 (thermal grease), 0.81 (vortex jet), 0.74 (preheating auxiliary), and 0.72 (direct injection) between the measured depth and hole deformation, respectively. The average coefficient of determination for all auxiliary mechanisms is 0.82. The relationship diagram shows that the correlation coefficient of the auxiliary and heat-dissipation-assisting mechanisms is the largest, whereas the correlation coefficient of the gas-assisted and preheating assistance is low. The slopes of the trend lines for four auxiliary mechanisms are similar, with the exception of the mechanism for preheating assistance. Under the same processing depth, the deformation around the holes is ordered from small to large: no auxiliary mechanism, thermal paste assist, swirl assist, and direct injection assist. The direct-injection-assisted gas is directly sprayed on the surface of the workpiece, which means that the heat dissipation effect is the fastest, and the temperature gradient is also the largest. The swirl assist also affects the surface of the workpiece. The gas pressure is small and the heat dissipation effect is second only to that of direct injection assistance, thus, the temperature gradient is the second largest. Using this diagram, it is easy to identify the mechanism that results in the most obvious temperature gradient, in addition to the part of the preheating experiment. The temperature of the workpiece changes significantly, which is why the slope of the trend line differs significantly for the various mechanisms. Figure 11 shows the actual image of the drilled hole.

## 4. Conclusions

In this study, digital image correlation (DIC) was combined with a non-traditional processing method to measure the residual strain. The main purpose was to develop a strain measuring system that is easy to establish and that is suitable for online testing. The experimental results can be summarized as follows. When the reference subset size is close to the spot size, displacement distribution data close to the actual situation can be obtained. The best matching subset size for Al 5052 is 51 × 51 pixels. Using the laser machining method to drill holes and observing the deformation near the hole, it was found that the area around the hole without the use of an auxiliary mechanism expands outward by about 1–2 μm, indicating that the temperature gradient generated during laser processing introduces thermal stress. This results in deformation around the hole. Furthermore, mechanisms using gas assistance and heat dissipation aggravate the temperature gradient, resulting in greater deformation. Finally, the pre-heated portion increases the deformation measured around the hole due to the thermal expansion of the workpiece. Linear regression showed an average value of the coefficient of determination of 0.82 between the measured depth and hole deformation among all auxiliary mechanisms. Using the above results, it was confirmed that DIC combined with the non-traditional processing method for strain measurement can be used with equipment that is simple and low-cost, namely, a tool microscope. This study proves that this method can be used for the measurement of small area deformation.

## Figures and Tables

**Table 1:** Experimental parameters of the laser.

Laser Pump Energy	Pulse Repetition Rate	Wave Length	Lens Focus Length
21 J	15 Hz	532 nm	120 mm

**Table 2:** Experimental parameters of air jet pressure.

ML-808 FX Dispenser	Rocker 300
300 kPa	21,000 Pa

**Table 3:** The maximum spot size obtained in three sets of samples.

Sample	1st	2nd	3rd
**size**	42 pixels	33 pixels	34 pixels

**Table 4:** Size of the reference subset (unit: pixel).

**Set**	**1**	**2**	**3**	**4**	**5**	**6**	**7**	**8**	**9**
**Size**	21	25	31	35	41	45	51	55	61
**Set**	**10**	**11**	**12**	**13**	**14**	**15**	**16**	**17**	-
**Size**	65	71	75	81	85	91	95	101	

## Data Availability

The data presented in this study are available on request from the corresponding author.

## Nomenclature

DIC	digital image correlation
ANN	Artificial Neural Network
*P*(*x*_0_, *y*_0_)	reference position by the center point
*P*′(*x*_0_′, *y*_0_′)	deformed position by the center point
*Q*(*x*_i_, *y*_j_)	reference position by the arbitrary point
*Q*’(*x*_i_’, *y*_j_’)	reference position by the arbitrary point
∆ε	difference between subsets
$\varepsilon_{M_{i}}$	strain of the displacement of subset *i*
∆D_x_	difference between the maximum and minimum deformations
